# Calreticulin (CALR)-induced activation of NF-ĸB signaling pathway boosts lung cancer cell proliferation

**DOI:** 10.1080/21655979.2022.2040874

**Published:** 2022-03-10

**Authors:** Fangfang Gao, Xiaoqian Mu, Huijuan Wu, Lijuan Chen, Jie Liu, Yanqiu Zhao

**Affiliations:** Department of Internal Medicine, Henan Cancer Hospital, Affiliated Cancer Hospital of Zhengzhou University, China

**Keywords:** CALR, lung cancer, NF-ĸB, proliferation

## Abstract

Calreticulin (CALR) is known to be aberrantly expressed in lung though the etiology underlying this phenomenon remains undetermined. The (Cancer Genome Atlas) databases were adopted to evaluate the expression status of CALR in pan-cancer, including Lung adenocarcinoma (LUAD) and Lung squamous cell carcinoma (LUSC) accompanied with Genotype-Tissue Expression project (GETx) database. Receiver operating characteristic (ROC) curves and Kaplan-Meier survival curve were plotted to assess its clinical significance in lung cancer. CCK8 and colony formation assays were conducted in addition to in vivo assays. The impact of CALR expression on NF-ĸB-mediated luciferase activity was detected by Luciferase assays. The regulatory relationship between CALR and NF-ĸB was further verified by NF-ĸB inhibitor treatment. LUAD and LUSC tissues reflected marked elevation in the mRNA levels of CALR. ROC analysis showed that CALR expression had a diagnostic value for LUAD or LUSC patients. High-CARL patients demonstrated inferior survival compared to that of Low-CALR patients. Functional assays revealed increased proliferative behaviors of A549 and H1299 cells associated with highly amplified while CALR gene inactivation could reduce the proliferation of both cells. CALR depletion decreased xenograft tumor growth. NF-ĸB transcriptional activity was found to be stimulated with CALR overexpression and reduced in CALR-deficient lung cancer cells, thereby clearly indicating CALR-dependent NF-ĸB activation. NF-ĸB specific inhibitors further validated enhanced NF-ĸB activity mediated by CALR overexpression. Conclusively, our results the role of CALR in lung cancer cells, indicating that highly expressed CALR proliferation at least by activation of NF-ĸB signaling pathway.

## GRAPHICAL ABSTRACT

**Figure:**
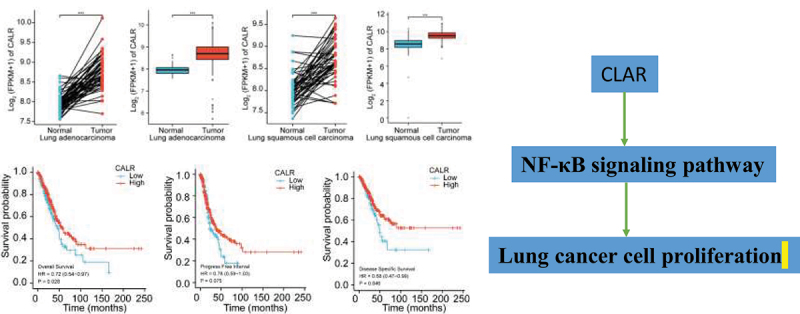

## Introduction

Lung cancer (LC) is a prevalent respiratory disorder persistent in both men and women. According to cancer statistics in 2020, with 228,820 new cases and 135,720 deaths in 2018 in it is listed as one of the four leading cancer types [1]. About 85% of LC are non-small cell lung cancer (NSCLC). Despite the improvement achieved in early diagnosis and treatment of LC, the disease outcome remains poor [2]. The role of gene mediation in LC carcinogenesis and progression has been documented [3,4]. Therefore, as far as LC diagnosis and therapy are concerned, complete elucidation of the underlying pathophysiology is crucial.

An endoplasmic reticulum (ER)-resident protein, is known to exhibit diverse physiological and pathological functions. It has been proved to evoke host immune response to preserve a dynamic balance between damaged cells and normal cells, signifying its role in antitumor immunity cancer. *In vivo* research with prostate cancer cells reported neoplastic transformation with downregulated CALR expression [5]. It is gaining considerable interest because its variant regulates immune cell infiltration and is associated with colorectal carcinoma [6]. Furthermore, prior investigations have reported the association of increased CALR expression with improved disease outcome of bladder cancer [7]. Nonetheless, the role of CALR in cancers is inconclusive considering the CALR gene regulating intracellular Ca^2+^ balance and integrin-dependent events [8]. For example, the risk of favorable prognosis in gastric cancers is enhanced with overexpression of CALR [9]. Currently, inconsistencies have also been documented in investigations into the prognostic value of CALR in lung cancer. Several reports have revealed the influences of robust CALR expression in NSCLC on the superior disease consequences of subjects [10–12], whereas Liu et al. reported contradictory results [13].

NF-ĸB signaling pathway is a key oncogenic element affecting carcinogenesis and progress [14]. In lung cancer cells, it is a dominant signal transduction pathway facilitating their oncogenic growth [15]. Potent antiapoptotic ability and sustained proliferative property during malignant transformation of normal lung cells are attributed to the NF-ĸB activation occurring simultaneously with K-Ras mutation [16]. Inhibition of NF-kappaB can reduce the secretion of inflammatory cytokines and improve the tumor microenvironment to suppress lung cancer progression [17]. Convincing evidence has substantiated that the of Ca^2+^ from the ER implicated NF-ĸB activation [18]. Therefore, we hypothesized that the activation of the NF-ĸB signaling pathway enhanced the traits of lung cancer cells. To evaluate the effect of CALR in lung cancer, we first examined the CALR expression in pan-cancer including LUAD and LUSC cohort. Based on the CALR expression profile from TCGA-LUAD and LUSC cohort, the prognosis and diagnostic accuracy were assessed. Gene set enrichment analysis (GSEA) was employed to analyze the CALR-related signaling events. Furthermore, we examined the CALR expression in the proliferation of A549 cells and H1299 cells by loss-gain function assays. The potential relationship between CALR expression and NF-κB oncogenic signaling pathway was also estimated in the present study.

## Methods

### Cells culture and reagents

The human NSCLC A549 cells, human lung adenocarcinoma H1299 cells, and human embryonic kidney HEK293T cells were purchased from the Cell Storage Center of Wuhan University (Wuhan, China). Cells were cultured in Ham’s F-12 k, RPMI-1640, and DMEM medium, respectively, at 37°C in a 5% CO_2_ incubator.

### Quantitative RT-PCR (qRT-PCR)

The RNA was isolated from 70% confluent lung cancer cells with the reagent (Sangon, China), and the purity was certified by measuring the ratio of OD260/OD280. A Reverse Transcription Kit (Takara, China) was used to reverse transcribe 1.0 µg RNA into first-strand cDNA. The QGreenTM 2X SybrGreen qPCR Master Mix (Thermo Fisher Scientific, China.) with specific primers was employed for the quantification of 2 µL of total cDNA. Each reaction was replicated three times. The data were normalized to the control transcript GAPDH with the 2^−ΔΔCt^ method. CALR forward primer: 5ʹACGATGAGGCATACGCTGAG3’, CALR reverse primer, 5ʹATCCACCCCAAATCCGAACC3’; GAPDH forward primer: GCAAATTCCATGGCACCGT; GAPDH reverse primer: TCGCCCCACTTGATTTTGG.

### Transfection

For CALR overexpression in A549 cells, the CALR cDNA sequences amplified by RT-PCR were inserted into pBabe-puro-3Xflag with EcoRI and BamHI restriction enzymes (Addgene, China). The constructed vectors named pBabe-puro-Flag-CALR and the empty vectors were transfected into sub-confluent 293 T cells following the standard calcium phosphate methods. After 24 h transfection, the obtained adenoviruses were infected with A549 cells of 50% confluence for 48 h. Subsequently, infected cells were subjected to 5-day puromycin selection, and stable CALR overexpression cell lines were obtained. The overexpression was validated by double-digestion with EcoRI/BamHI as well as Western blot assay.

CALR expression was knocked out in A549 and H1299 cells using the CRISPR/Cas9 system. Firstly, two sgRNAs targeted for exon 1 and exon 3 of CALR (sgRNA1 CCTCTAATCCCCCACTTAGACGGGTGGACT; sgRNA2: TCACCAACGATGAGGCATACGCTGAGGAGT;) were designed via the E-CRISP sgRNA design tool and synthesized by Wuhan Tianyi Co. Ltd, China. The sequence of sgRNA was linked into pSD-gRNA (Biomics Biotech, China) to generate sgRNA intermediary vectors (hereafter called CALR sgRNA). The CALR sgRNA and the pXT7-hCas9 (Addgene, China) were co-transfected in H1299 cells for 48 h. Finally, ampicillin plates containing 100 µg/mL of ampicillin were used to screen the positive clones, which were subjected to validation by Western blot.

### Western blot

Monocytes were lysed by sonication and centrifuged for 3 min. The supernatant was collected, and the protein dose was estimated by a BCA The protein assay kit (ThermoFisher, China). Protein samples (20 µg) were electrophoretically separated by 10% SDS-PAGE and bolted to PVDF membranes. Before the blot was blotted against anti-CALR (RK-100-401-E99, 1:1000, Rockland, USA), anti-GAPDH (ab8245, 1:1000, Abcam, USA), anti-Flag (ab93713, 1:1000, Abcam, USA), or anti-IĸBa (ab32518, 1:1000, Abcam, USA) at a temperature of 4°C, the membranes were subjected to blockage with 2% nonfat dry milk at room temperature. The next day, anti-mouse IgG secondary antibodies (ab202646, 1:1000, Abcam, USA) were added, and the membranes were incubated at 37°C for 3 h. The signals were visualized with the help of an ECL Plus kit (Haimen, China).

### Cell proliferation assay

The cell proliferation was detected by a CCK8 kit (Dojindo, Shanghai, China). Briefly, 5000 cells/well were seeded in 96-well plates and cultured for 1, 3, and 5 days before 10 µL CCK8 agent was supplemented. After another 1 h, the OD value was measured at 450 nm by a microplate reader (Promega, China).

### Colony formation assay

The cells (n = 1000) suspended in a fresh medium containing FBS were plated in 6-cm plates and cultured for 12 days. After obtaining the colonies >50 nm, the supernatant was discarded, and the plates were exposed to 4% formaldehyde for 15 min prior to staining with GIMSA for 15 min. The colony number was recorded via an inverted microscope.

### Tumor growth analysis

Twelve nude mice, aging 4–6 weeks and weighting about 20 g, were bought from the Experimental Animal Center of Wuhan University (Wuhan, China). Mice were kept in a condition with appropriate, and light conditions. Water and food were available at all times. The Animal Research Committee of XX hospital was approve the animal assays. H1299 cells (8 × 10^6^) with CALR knockout or normal H1299 cells (8 × 10^6^) were administered subcutaneously in flank of nude mice. On day 28, mice were killed with CO_2_ and tumors were and weighted.

### Bioinformatics and statistical analysis

Bioinformatics analysis was performed using R (R version 3.6.3) and visualized with ggplot2. CALR expression data (HTSeq-FPKM) in TCGA cohort pan-cancer database and was acquired from TCGA (https://portal.gdc.cancer.gov/) along with the clinical information of LUAD and LUSC patients. Differences between non-paired specimens were examined with Wilcoxon rank sum test, and differences between paired specimens with paired-test. The downloaded TCGA-LUAD and TCGA-LUSC data were also applied to assess the diagnostic and prognostic value of CALR in LUSC and LUAD by receiver operating characteristic (ROC) curves.

Other statistical parameters were evaluated via the GraphPad Prism 7.0 software package. Three repeated data were represented as mean ±SEM. One-way analysis of variance (ANOVA) with Tukey’s post hoc test was used to analyze the data of multiply groups. An unpaired t-test with the Mann-Whitney test was utilized to assess the data of two groups. *P* < 0.05 is assigned as a significant difference.

## Results

After a series of bioinformatics analysis of CALR in lung cancer, we further explored CALR-mediated activation of the NF-ĸB signaling pathway in lung cancer progression.

### CALR mRNA level is elevated in lung cancer

**Figure 1.**
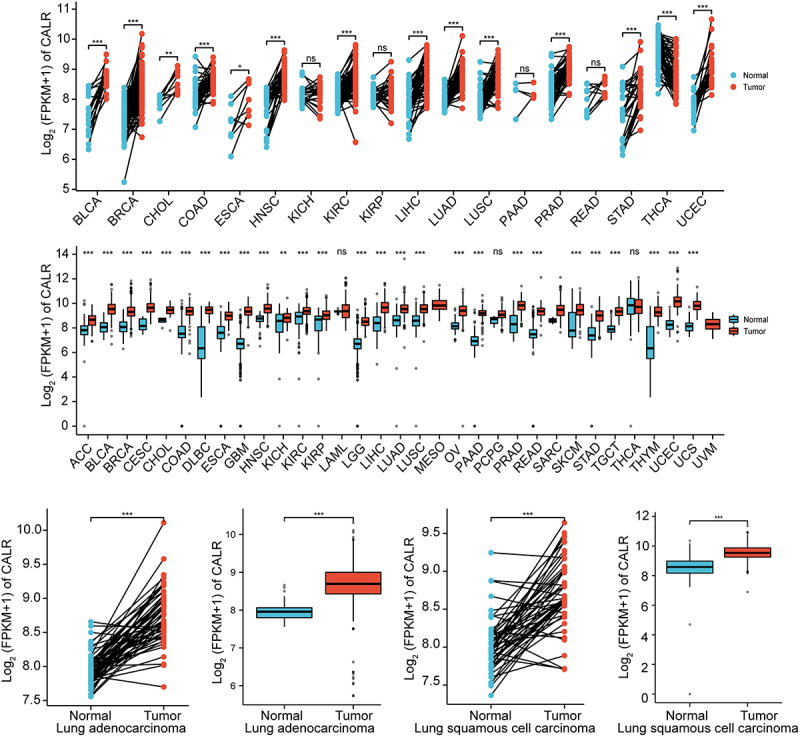
(a). The expression of CALR in pan-cancer and corresponding normal tissues derived from TCGA database. (b) The expression of CALR in pan-cancer and corresponding normal tissues derived from TCGA database in addition to normal lung tissues derived from GETx database. (c). The expression of CALR in TCGA-LUAD database and matched normal tissues. (d). The expression of CLAR in TCGA LUAD tissues compared to that of TCGA and GETx normal lung tissues. (e). The expression of CALR in TCGA-LUSC database and matched normal tissues. (f). The expression of CLAR in TCGA LUSC tissues compared to that of TCGA and GETx normal lung tissues.

**Figure 2.**
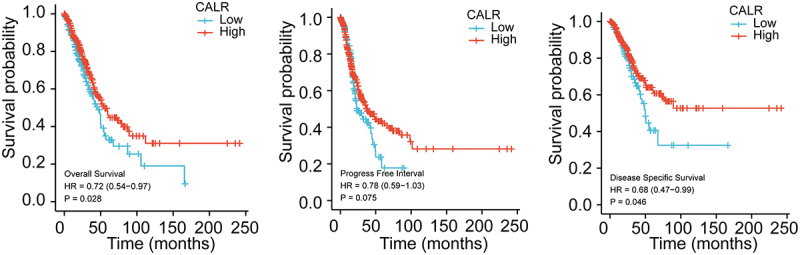
Assessment of CALR prognostic value during lung cancer progression. (a). Overall survival association for low and high CALR mRNA level from TCGA-LUAD and TCGA-LUSC cohort. (b). Progress free interval for low and high CALR mRNA level in TCGA-LUAD and TCGA-LUSC cohort. (c). Disease specific survival for low and high CALR mRNA level in TCGA-LUAD and TCGA-LUSC cohort.

Previous evidence recognized the involvement of CALR in lung cancer. Henceforth, the CALR mRNA level was evaluated in TCGA database in addition to GETx database. We first analyzed the CALR expression in pan-cancer based on the TCGA database. Compared with the matched normal tissues, the highly expressed CALR was detected in different types of tumors (TCGA tumor vs TCGA normal), including LUAD and LUSC (Figure 1(a)). In comparison with GTEx and TCGA normal, the robust expression of CALR was also witnessed in TCGA cancer samples, including LUAD and LUSC (Figure 1(b)). Reanalyzing RNA-seq data downloaded from TCGA-LUAD and TCGA-LUSC, we observed upregulated CALR levels in LUAD tissues compared with those of paired non-malignant lung tissues and those of non-malignant lung tissues (TCGA normal + GTEx normal), respectively (Figure 1(c,d)). Likewise, we also analyzed the CALR expression in LUSC tissues based on TCGA and GTEx database. Undoubtedly, CALR amplification was detected in LUSC tissues (Figure 1(e,f)). Subsequent prognostic analysis showed that high-CALR patients exhibited low overall survival (Figure 2(a)), shorter progress free interval (Figure 2(b)) and poor disease specific survival (Figure 2(c)) compared with low-CALR patients.

### Diagnostic value of CALR expression in lung cancer

**Figure 3.**
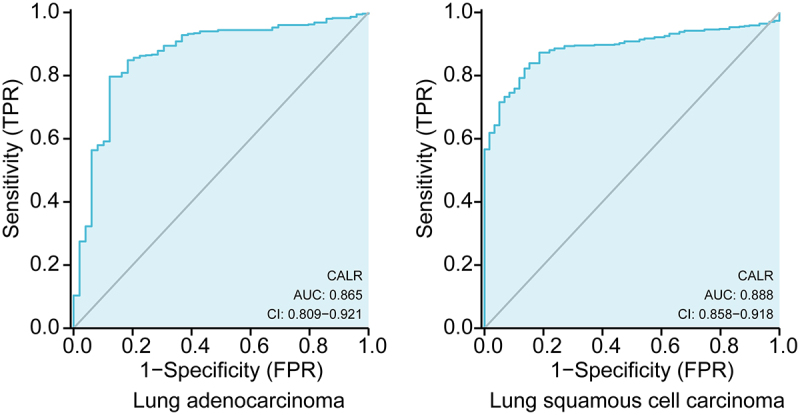
ROC curve (AUC) of CALR in the diagnosis of LUSC and LUSD data from TCGA database.

Subsequent ROC analysis was performed to verify the diagnostic significance of CALR expression according to the clinical information acquired from TCGA database. As showed in Figure 3(a,b), the area under the curve (AUC) was 0.865 (95% CI: 0.809‐0.921; P < 0.0001) for LUSC patients, 0.888 (95% CI: 0.858–0.918; P < 0.0001) for LUAD patients. All these results demonstrated that CLAR expression might precisely discriminate tumor from non-tumor tissues.

### Identification of CALR-associated signaling pathway by GSEA

**Figure 4.**
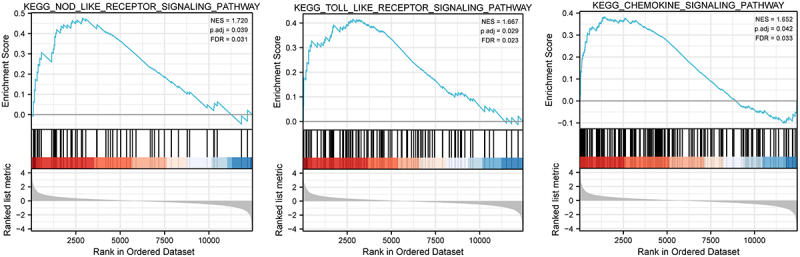
Gene set enrichment analysis (GSEA) was used to compare gene expression profiles related high-CALR and low-CALR. FDR, false discovery rate; NES, normalized enrichment score.

Next we further characterized the biological pathways impacted by CALR expression based on the database from TCGA-LUAD and TCGA-LUSC cohort. In addition to adjust p value, the normalized enrichment score (NES) and false discovery rate (FDB) were applied to assort the CALR-associated signaling events. As depicted in Figure 4(a) and Table 1, KEGG nod like receptor signaling pathway, KEGG toll like receptor signaling pathway and KEGG chemokine signaling pathway showed positive linkage with CALR mRNA levels in these TCGA cohort.

**Table 1.** Gene sets enriched in the high CLAR expression phenotype

Gene set name	Size	ES	NES	p. adj	FDR
KEGG_NOD_LIKE_RECEPTOR_SIGNALING_PATHWAY	56	0.477	1.720	0.0039	0.031
KEGG_TOLL_LIKE_RECEPTOR_SIGNALING_PATHWAY	97	0.416	1.667	0.0029	0.023
KEGG_CHEMOKINE_SIGNALING_PATHWAY	166	0.383	1.652	0.0060	0.033

### *Overexpression of CALR promotes proliferation and colony formation* in vitro

**Figure 5.**
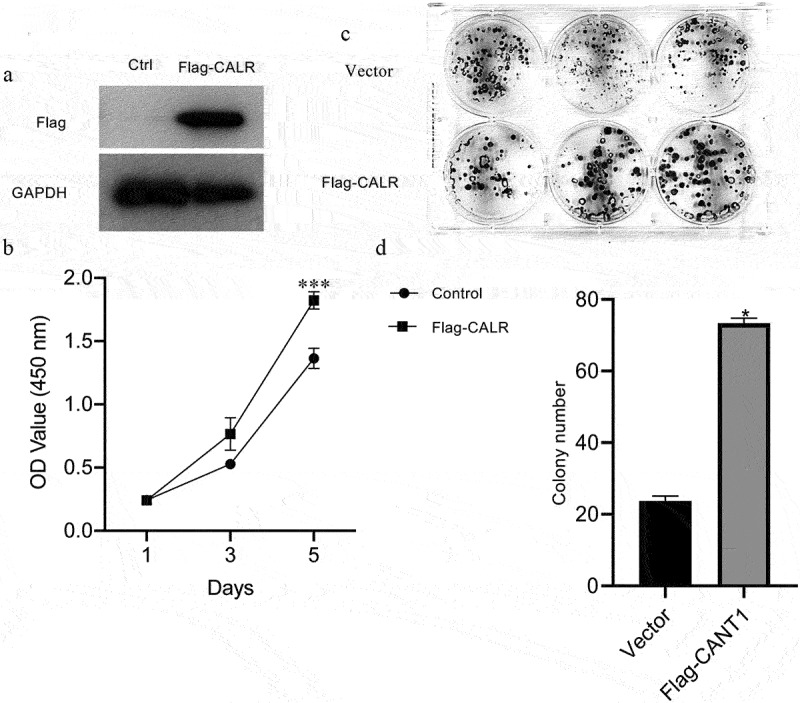
Overexpression of CALR promotes the proliferation and colony formation under *in vitro*. a. Western blot demonstrating CALR expression in A549 cells. GAPDH serves as a loading control. b. CCK8 of A549 cells after infection with CALR-overexpressing vectors and its empty control vectors. c and d. Colony formatting assay for the A549 cells after infection with CALR-overexpressing vectors and its empty control vectors.

To elucidate the role of CALR in the proliferation of lung cancer cells, CALR was overexpressed by infecting A549 cell lines with CALR-overexpressing plasmids. Western blot assays validated the overexpression (Figure 5(a)). The highly expressed CALR resulted in an augmentation of the proliferative capacity (Figure 5(b)). The CALR overexpression was further associated with a conspicuous increase in colony formation of A549 cell lines (Figure 5(c,d)).

### CALR depletion blunts lung cancer growth

**Figure 6.**
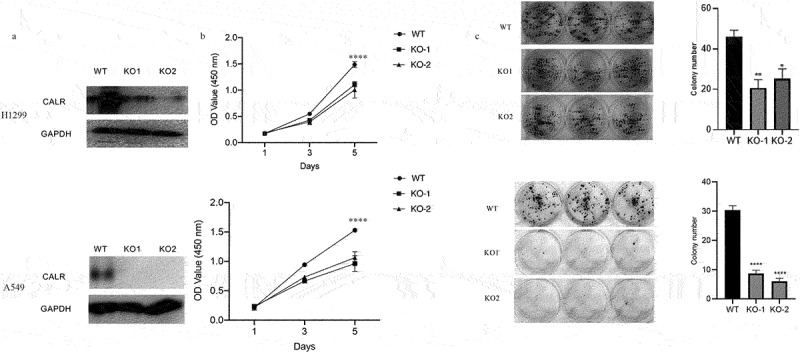
CALR depletion decreases the proliferation and viability of H1299 cells (a). Western blot of CALR following transfection using lentiCRISPR v2-sgRNA-CALR or its empty plasmids to H1299 and A549 cells. (b). CCK8 assays for the assessment of CALR depletion during H1299 and A549 cell proliferation and (c) and (d) Colony formation to clarify the effects of CALR depletion on H1299 and A549 cell colony formation.

**Figure 7.**
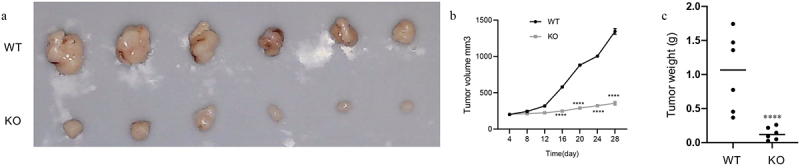
The effect of CALR depletion on xenograft tumor growth. (a). Representative xenografts dissected from different groups of nude mice were shown. (b) and (c). Tumor volume and weight were examined in xenograft tumor that was formed by CLAR-depleted H1299 cells and wide-type H1299 cells.

To further define the biofunctional role of CALR in LC progression, we developed two knockout clones (KO-1 and KO-2) (Figure 6(a)) to study the effects of CALR depletion on proliferation and colony formation of H1299 and A549 cells. As illustrated in Figure 6(b), CALR depletion negatively affected the proliferation of H1299 and A549 cells. This was further substantiated by the CALR depletion-mediated reduction in colony formation of H1299 and A549 cells (Figure 6(c,d)). Further *in vivo* assays demonstrated that CALR depletion inhibits tumor growth (Figure 7). Collectively, our findings proposed that CALR depletion could decrease LC growth *in vitro* and *in vivo*.

### CALR triggers NF-ĸB signaling pathway

**Figure 8.**
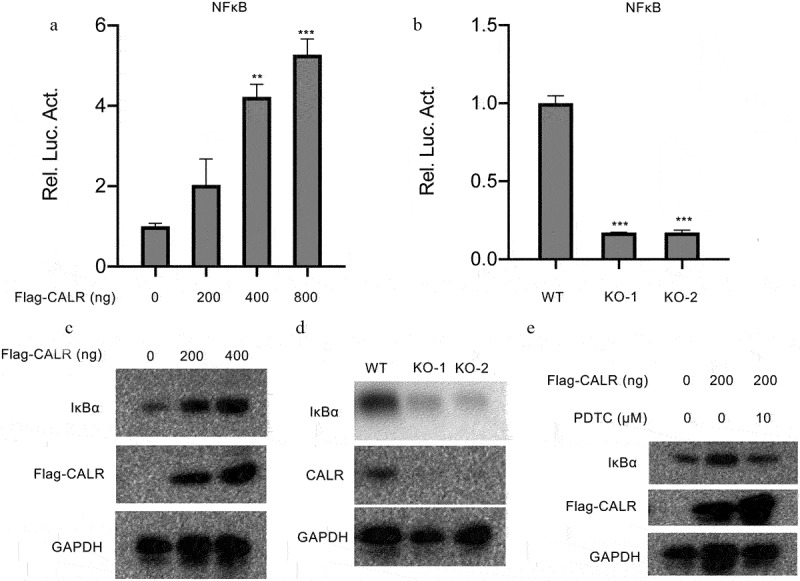
CALR activates NF-ĸB signaling pathway in lung cancer cell lines. (a). the NF-ĸB transcriptional activity in the A549 cells transfected with overexpressing CALR vectors. (b). the NF-ĸB transcriptional activity in H1299cells. (c), (d) and (e). IĸB expression levels in the CALR overexpressing A549 cells transfected with vectors, in CALR depleting H1299 cells and in CALR overexpressing A549 cells exposed to 10 µm PDTC.

TOLL-like receptors, NOD-like receptors and chemokine signaling pathway are vital for inflammation during tumor genesis and cancer progression. Therefore we examined the NF-ĸB signaling pathway which which is implicated in regulation of inflammatory response Therefore luciferase reporter assays were conducted to reveal the interactions between CALR and NF-ĸB signaling pathway. Intriguingly, our results demonstrated dose-dependent stimulation of NF-ĸB-mediated luciferase activity in A549 cells induced by CALR overexpression (Figure 8(a)). Consistently, a significant reduction of NF-ĸB-mediated luciferase activity was evident in the two KO H1299 cell clones compared with the control cell lines (Figure 8(b)). These data claimed that CALR NF-ĸB downstream transcription events. Furthermore, we also explored the expression of IĸB, an NF-ĸB-responsive downstream gene. The results indicated the accumulation of IĸB protein in CALR overexpressing A549 cells (Figure 8(c)), whereas attenuation in CALR deficient H1299 cells (Figure 8(d)). Inactivation of NF-ĸB signaling pathway by an NF-ĸB inhibitor could curb the increased IĸB expression caused by CALR overexpression (Figure 8(e)). These findings, therefore, concluded the role of CALR/NFĸB axis in lung cancer progression.

## Discussion

Research findings substantiated a relevant correlation between lung cancer progression and clinical outcome with CALR abnormal expression [10–12]. However, the physiological and pathological mechanisms remain completely untangled. In our current work, we investigated the effect of CALR expression level on lung cancer cell proliferation and its underlying mechanism. Bioinformatics analysis illustrated upregulation of CALR expression in pan-cancer cohort, including LUAD and LUSC. The parallel analysis illustrated that CALR mRNA levels had diagnostic and prognostic values during lung malignancy. Furthermore, *in vitro* functional assays claimed that CALR overexpression favored the proliferation and colony formation of A549 cells. Contradictorily, its deficiency impaired the proliferation *in vitro* as well as inhibited tumor growth *in vitro* and *in vivo*. Mechanically, the NF-ĸB signaling pathway was found to be involved in CALR-regulated lung cancer progression. Overall, CALR has a potential promoting role in the advancement of lung carcinoma.

Investigations revealed the involvement of CALR in diverse types of malignancy, including lung cancer [5,12,19,20]. Hence we first analyzed the CALR expression in pan-cancer based TCGA and GETx database. The findings demonstrated it robust expression in different malignancies, including LUAD and LUSC. However, its role and prognostic values in lung cancer appear to be contradictory. In three independent cohorts of subjects suffering from NSCLC, histopathological examination detected low CALR level, which was related to the unfavorable disease outcome [21,22]. Moreover, Stoll et al. (2016) demonstrated that suppressed antitumor immune surveillance mediated by reduced CALR expression might negatively impact the prognosis of NSCLC patients [11]. In sharp contrast, Liu’s (2016) recorded elevated expression of CALR in NSCLC patients resulted in the dismal prognostic outcome [13]. Our present data revealed unregulated CALR mRNA levels based on TCGA database. More importantly, poor inferior prognosis was witnessed among high-CALR patients. In addition, the CALR deferential expression in lung cancer was valuable in lung cancer diagnosis. All these data underscored the involvement of CALR expression during lung tumorigenesis and progression.

Imbalance of ER Ca^2+^ homeostasis leads to increased proliferation of lung cancer cells, and the consequence of increased Ca^2+^ production seemed to trigger apoptosis of A549 cells when exposed to ER stress [23]. On the other hand, CALR was reported as an essential modulator of Ca^2+^ homeostasis [23]. To further dissect the effect of CALR expression on functions of lung cancer cells, we overexpressed CALR in A549 cells and knocked out CLAR in A549 and H1299 cells. The proliferation assays results claimed escalation of proliferative phenotypes of A549 cells associated with CALR overexpression whereas reduced viability of H1299 and A549 cells in case of CALR gene inactivation. Our in vivo assays further demonstrated that CALR depletion impaired the tumorgenesis of lung cancer. Our results were in agreement with Zheng’s report illustrating CALR expression operates as an oncopromoter in T-cell lymphoma [8]. At present, the function of the CALR gene on lung cancer cell invasion and metastasis is yet to be studied.

To further understand the mechanical role of CALR in lung cancer, we adopted the TCGA-LUAD and TCGA-LUSC cohort to sort the dysregulated signaling cascades during lung malignancy by GSEA analysis. The results demonstrated the signaling of NOD like receptor, TOLL like receptor and chemokine were enriched. Convincing evidence has recorded these three signaling events are generally activated during innate immune and inflammatory responses and showed a tight association with NF-ĸB signaling pathway. Overactivation of the NF-ĸB signaling cascade is frequently witnessed in lung cancer and stimulates cell proliferation. Its potentiation is associated with ER Ca^2+^ homeostasis [24], which, is reported to be regulated by CALR [8]. Therefore, we explored the involvement of CALR with NF-ĸB activation. The present study substantiated increased NF-ĸB-mediated luciferase activity in A549 cells infected with different concentrations of CALR-overexpressing vectors. Reduced NF-ĸB transcriptional activity was also manifested in CALR-deficient H1299 cells. Modulation of IĸBa expression in association with CALR expression further validated the NF-ĸB role in lung cancer. These findings indicated a CALR-mediated alteration of NF-ĸB signaling in lung cancer. PDTC a specific inhibitor of NF-ĸB, was found to suppress IĸB degradation and downregulate NF-ĸB events. Treatment with PDTC could significantly diminish the increased IĸBa expression induced by CALR overexpression. Our findings, thus, corroborated the hypothesis that CALR modulated NF-ĸB events in lung cancer. However, in contradiction with our results, Liu et al. (2015) documented that the enforced expression CALR suppressed the carcinogenesis of NSCLC by inactivation of NF-ĸB signaling events in A459 cells. This, in turn, triggered antigen-specific T cell immunity, thereby resulting in an anticancer effect to NSCLC [13]. These challenging results deserve further validation.

## Conclusion

Overall, our present results indicate that CALR activates NF-ĸB signaling in lung cancer cells and increase cancer cell proliferation. These findings affirm the significant role of CALR in the progression of lung carcinoma.
